# *BMC Blood Disorders* becomes *BMC Hematology*: evolving along with the hematology field

**DOI:** 10.1186/2052-1839-13-1

**Published:** 2013-04-10

**Authors:** Christna Chap

**Affiliations:** 1 BMC Hematology, BioMed Central, 236 Gray’s Inn Road, London, WC1X 8HB, UK

## Abstract

This Editorial marks the launch of *BMC Hematology,* formerly known as *BMC Blood Disorders,* within the *BMC* series of journals published by BioMed Central. The scope of *BMC Hematology* encompasses basic, experimental and clinical research related to hematology. In this Editorial we will discuss the rationale behind this relaunch and how, as an open access journal providing unrestricted and free access to scientific and scholarly work*, BMC Hematology* will help disseminate research in the hematology field in a freely-accessible manner.

## Editorial

### Aims and scope

To emphasize its commitment to representing the full breadth of disciplines and specialties in hematology, *BMC Blood Disorders* has relaunched this month under the name *BMC Hematology*. The decision to rename the journal was taken in response to the feedback received from key opinion leaders in the field and in consultation with our Editorial Board.

This change of journal name is also intended to reflect the multi-disciplinary nature of the hematology field in which rapidly evolving research findings translate into patient care. Since William Harvey introduced the then controversial concept of circulation in 1628, the field of hematology has come a long way. Important advances in the field of molecular biology have enabled fundamental insights into the origin of human diseases and enabled the development of innovative treatments. Nowhere are these changes more prominent than in the field of hematology, which has indeed paved the way for the thrilling era of “bench to bedside” research. The past decades have seen major clinical advances arising from trail-blazing basic research, including hematopoietic stem cell transplantation for the treatment of blood and bone marrow disorders and targeted molecular therapies for hematological malignancies. Reflecting the cross-disciplinary and translational nature of the field, *BMC Hematology* welcomes submissions on basic, experimental and clinical research related to non-malignant and malignant hematological diseases, hemostasis and thrombosis, hematopoiesis, stem cells and transplantation.

*BMC Hematology* will maintain the ethos of its predecessor and of the rest of its sister journals in the *BMC* series [1], and continue to publish work deemed by peer reviewers to be a coherent and sound addition to scientific knowledge. There will be less emphasis on interest levels, provided that the research constitutes a useful contribution to the field [2]. All previous content will remain freely accessible from the new journal homepage.

Similarly to other journals in the *BMC* series, *BMC Hematology* has an international Editorial Board of academic scientists, many of whom are previous members of *BMC Blood Disorders*[3]. The journal is divided into a number of subject-specific sections under the oversight of Section Editors, who are supported by Associate Editors and Editorial Advisors as well as in-house Editors. Dividing the journal into sections not only allows authors to submit to a specific section but also enables readers to browse articles in a given area of interest to them [4].

As with *BMC Blood Disorders* and all other medical journals within the *BMC* series, *BMC Hematology* will operate an open peer review policy with two levels to this “openness” [5]. The first is that authors will see the reviewers’ names; the second is that the reading public will also see who reviewed the article and how the authors responded, if the article is published. This will be available as part of the pre-publication history of the published article. The most important benefits of openness are accountability, fairness and giving credit to reviewers for their efforts. By making the process open, we also aim to reduce the competing interests that can occur during peer review [6,7].

### Open access

Although initially looked upon with skepticism, the open access publishing model, which provides an unrestricted and free access to scientific and scholarly work, has become increasingly accepted as a viable model, with more than 8,500 open access journals now operating worldwide [8].

Open access increases the potential readership of any article to anyone with internet access and indirectly accelerates the spread of new research findings. All articles published in *BMC Hematology* are included in the major bibliographic databases including PubMed and can be freely redistributed and reused as long as they are correctly attributed. Publication volume has grown within all major scientific disciplines, but biomedicine has seen a particularly rapid 16-fold growth between 2000 (7,400 articles) and 2011 (120,900 articles) [9]. Approximately 17% of all articles published in 2011 are open access. In a decade, this model has moved from the fringe to the core of the publishing industry.

Articles published in *BMC Hematology* will of course be open access, ensuring that research is disseminated to the widest possible audience. In order to cover the costs of publication, an article-processing charge (APC) is levied to the authors or their institution on acceptance of the article. This flat fee is among the lowest charged across other publishers offering open access [10]. Publishing in electronic format allows the full use of digital technologies and permits the inclusion of large data sets or video clips for instance, and unlimited color and page numbers at no additional charge. If the institute of the authors submitting a manuscript is a member of BioMed Central, the cost of the APC is covered by the membership and no further charge is payable [11]. A number of funding agencies allow their grants to be used for APCs [12] and waivers can be granted under special circumstances. Following in the footsteps of the National Institutes of Health in the USA, public research funders in the UK have recently published guidelines to encourage and increase open access to publicly funded research [13,14].

### Looking ahead

We are delighted to welcome our two first Section Editors Dan L. Longo from Harvard Medical School and Mary J. Laughlin from University of Virginia, heading the Malignant hematological diseases and Hematopoiesis, stem cells and transplantation sections respectively. We plan to recruit additional Editorial Board members from many different disciplines within the hematology field as well as from different geographic regions. We believe that a strong Editorial Board is essential to ensure that the journal is at the forefront of the field while effectively using the time and effort that the reviewers contribute. In this inaugural issue, Section Editor Dan L. Longo tells us how he first became interested in hematology and gives his personal view on the recent progress and future challenges of the hematological cancer field in particular [15]. The relaunch of *BMC Hematology* marks the start of an exciting new developmental phase for the journal and we do hope that you will take the time to visit the website and consider us for your future submissions.

## Competing interests

CC is the Executive Editor of *BMC Hematology*.

## Pre-publication history

The pre-publication history for this paper can be accessed here:

http://www.biomedcentral.com/2052-1839/13/1/prepub
